# Patients’ adherence to optimal therapeutic, lifestyle and risk factors recommendations after myocardial infarction: Six years follow-up in primary care

**DOI:** 10.1371/journal.pone.0202986

**Published:** 2018-09-04

**Authors:** Clarisse Dibao-Dina, Denis Angoulvant, Jean-Pierre Lebeau, Jean-Eudes Peurois, Karim Abdallah El Hirtsi, Anne-Marie Lehr-Drylewicz

**Affiliations:** 1 General Practice Department and INSERM SPHERE U1246, University of Tours, Tours, France; 2 Cardiology Department and EA4245, University of Tours, Tours University Hospital, Tours, France; 3 General Practice Department and EA EES, University of Tours, Tours, France; Azienda Ospedaliero Universitaria Careggi, ITALY

## Abstract

**Background:**

The 20% observed mortality within 5 years among survivors of myocardial infarction may be explained in part by non-adherence to the recommended treatment over the long term.

**Main objective:**

To investigate post-myocardial infarction patients’ adherence to the therapeutic, lifestyle and risk factor control recommendations of the French health authority over 6 years.

**Materials and methods:**

A cohort of survivors of myocardial infarction established in Tours in 2009 was followed over 6 years. The general practitioner of the patients included in the first 1-year follow-up study was contacted to collect data on treatments, cardiovascular risk factors and lifestyle between January and June 2015. Data were described and compared with the recommendations and predictors of achieving all the recommended targets were determined.

**Results:**

A total of 97 patients (52%) among the 185 patients who underwent a coronary angioplasty for MI were included at baseline. Treatment was adapted by the general practitioner on the advice on the cardiologist for 75% (73/97) patients, by both of them for 12% (12/97) and by the general practitioner alone for 7% (7/97) patients. Among the 97 initial patients, 62 were included in the final analysis at 6 years. Fatal events rate was 5% (5/97) at 1 year and 12% (11/91) at 6 years. Non-fatal events rate was 44% (43/97) at 6 months, 19% (17/91) at 1 year and 29% (18/62) at 6 years. Six years after the myocardial infarction, 6 (10%) patients achieved the recommended targets in terms of prescriptions of treatment, risk factors and lifestyle targets. Exposure to a cardiac rehabilitation program after a myocardial infarction was associated with long-term achievement of optimal therapeutic objectives (OR = 7.31 [95% CI 1.74; 44.88], p<0.002).

**Conclusion:**

Our data show suboptimal long-term adherence to secondary prevention treatment in this high-risk population of survivors of myocardial infarction, which seems to be improved in patients exposed to a cardiac rehabilitation program.

## Introduction

Survival of patients with non-ST-elevation myocardial infarction (NSTEMI) significantly improved between 1995 and 2010 in France [1]: the rate of death at 1 year decreased from 20% in 1995 to 9.8% in 2010 (adjusted hazard ratio 0.49 [95% CI 0.38; 0.63]) [1]. Determinants of this decrease included a more invasive strategy with early percutaneous coronary intervention, the use of anticoagulants rather than unfractionated heparin and the early use of antiplatelet agents, beta-blockers, angiotensin-converting enzyme inhibitors (ACEIs) and statins [1].

However, long-term survival after a myocardial infarction (MI) has not improved. In the United Kingdom and Belgium, long-term prospective registries demonstrated an increase in mortality among MI survivors between the initial hospital phase (3% to 4%) and 5-year follow-up (15% to 18%) [2].

To reduce cardiac morbidity and mortality, international guidelines recommend lifestyle interventions, risk factors control and treatment combining beta-blockers, aspirin/clopidogrel, statins and ACEIs or angiotensin receptor blockers (ACEIs/ARBs) after an MI [3]. However, non-adherence to evidence-based treatment was found to be frequent and adversely affect long-term survival [4–5]. A way to improve the cardiovascular risk post-MI would be to increase the collaboration between the specialties of cardiology and general practice, and between hospital and outpatient settings [6–7].

The majority of studies on long-term management post-MI evaluated outcomes from 6 to 30 months after the MI [4], [6–10]. However, the aim of secondary prevention is to decrease the mortality or risk to get a new cardiovascular event at 5 to 10 years. Amman et al evaluated long term survival > 5 years post-MI, but it was only on treatments’ recommendations and based on data from registries, prone to memory bias [11].

As a result, a study on the long-term management > 5 years post-MI in outpatient’s setting is lacking. The aim of our study was to investigate the long-term adherence of post-MI survivors to evidence-based treatment, lifestyle and risk factors objectives 6 years after a MI according to the recommendations of the French health authority in a primary care setting [12].

## Materials and methods

This was a prospective cohort study. Patients who underwent a coronary angioplasty for MI in the university hospital centre of Tours between January and June 2009 were recruited. We included patients with an acute coronary syndrome, with ST-segment elevation and/or without ST-segment elevation with elevated troponin level, and excluded those without administrative data, who underwent a coronary angioplasty for diagnosis, who died before discharge or who were followed only by the university hospital centre.

Data were extracted from patient records at the hospital and included age, name of general practitioner, date of MI, presence of cardiovascular risk factors (arterial hypertension, dyslipidemia, diabetes, smoking status), biology findings in the initial phase (glycaemia and levels of low-density lipoprotein cholesterol [LDL-cholesterol] and glycated hemoglobin [HbA1C] for diabetic patients) and left-ventricular ejection fraction (for ACEI indication). Initial management of the MI was also reported, specifying the presence of a stent or coronary bypass surgery and medical treatment at discharge.

Data were completed by questionnaires sent to patients’ general practitioners at 6 months and 1 year to assess maintenance of therapeutic, lifestyle and risk factors control recommendations [12]. The recommended treatment included the “BASI” treatment (B for beta-blocker, A for platelet aggregation inhibitor, S for statin and I for ACE inhibitor) [12]. The type of prescribed molecule and doses for beta-blockers and ACEIs/ARBs were also reported and compared with the recommended ones. The lifestyle recommendations included tobacco cessation and regular physical activity [12]. The recommended risk factors management included the achievement of LDL cholesterol (<1g/L) and arterial blood pressure (<140/90 mmHg) targets for all patients and HbA1C (<6.5%) targets for diabetic patients [12]. Other targets for LDL cholesterol (<0.7g/L and <0.8g/L) were detailed according to European recommendations [3]. Type of follow-up by a general practitioner and/or a cardiologist in private practice was described. Non-fatal cardiovascular events were also reported. At 6 years, the questionnaire was completed by reporting fatal and non-fatal cardiovascular events, morbidity interfering with the recommended treatment post-MI, physical activity (at least once a week of biking, swimming, running or walking more than 1 or 2 hours) and participation in a cardiac rehabilitation program.

Data were collected through a standardized form consensually created by all the authors and tested by 3 independent general practitioners who were not involved in the study (neither as a participant nor as an author). The questionnaire was then administered by phone or face to face by two specifically trained researchers (KA & JEP). Whenever a general practitioner could not fill all the sections at once, he could complete the missing responses by either referring to his patient’s folder or calling the patient. In this last eventuality, he had to first collect the written informed patient’s consent. The primary outcome was the achievement of all therapeutic prescriptions (i.e. prescription of the BASI treatment), lifestyle (i.e. tobacco cessation and regular physical activity with at least once a week of biking, swimming, running or walking more than 1 or 2 hours) and risk factor control recommendations (i.e. achievement of LDL-cholesterol and arterial blood pressure recommended targets for all patients and HbA1c targets for diabetic patients as described above). Secondary outcomes were each recommended outcome analyzed separately (i.e. each therapeutic class of BASI treatment, tobacco cessation, physical activity and each risk factor target).

All analyses involved using SPSS v18.0 (SPSS Inc., Chicago, IL, USA) with two-sided P<0.05 considered statistically significant. Categorical data are described with frequencies (percentages) and quantitative data with mean ± SD. Categorical data were compared by chi-square test or, as appropriate, Fisher exact test. To determine predictors of achieving all the recommended targets, we used univariable logistic regression analysis of the outcomes adherence to treatment (BASI), lifestyle (tobacco, physical activity) and risk factor control (LDL-cholesterol, arterial blood pressure and HbA1C) recommendations, according to age, cardiac rehabilitation and type of follow-up. Data are presented as odds ratios (ORs) with associated 95% confidence intervals (CIs).

Any aspect of the work covered in this manuscript that involved human patients has been conducted with the ethical approval of the National Commission for Data Protection and Liberties (CNIL-France) (no. 1899240), according to the ethical committee IORG0008143 OMB No. 0990–0279. The data were analyzed anonymously.

## Results

**Fig 1:**
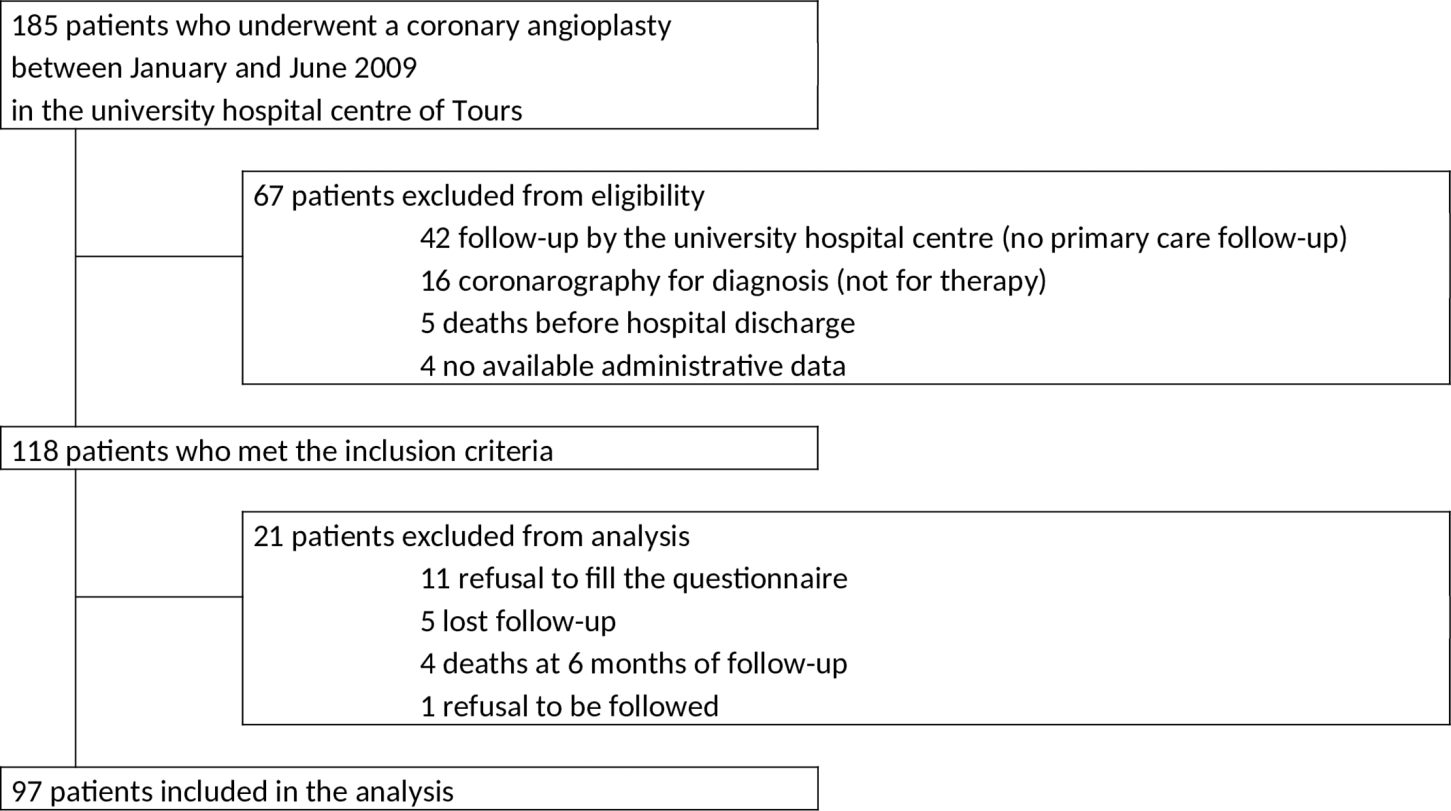
Flowchart.

A total of 97 on the 185 patients who underwent a coronary angioplasty between January and June 2009 were included in the analysis (Fig 1).

### Population characteristics

The characteristics of the 97 analyzed patients are presented in Table 1. At 1 year, 5/97 (5.2%) had died and 1 (1.0%) patient was lost to follow-up. At 6 years, 11/91 (12.1%) had died and 18/91 (19.8%) were lost to follow-up. Proportions of adherence to recommended targets and death are presented in Table 2. Results about survival at 1 year and 6 years are presented in Table 2 according to adherence rates to recommended targets on previous evaluations at 6 months and 1 year respectively.

**Table 1:** Baseline characteristics of patients with myocardial infarction at hospital discharge (n = 97).

Characteristics		
Age (years), mean±SD		67.6 ± 12.5
Gender (male)		67 (69.1%)
Cardiovascular risk factors		
	Arterial hypertension	65 (67.0%)
	Diabetes	20 (20.6%)
	Active smoker	23 (23.7%)
	Dyslipidemia	56 (57.7%)
	BMI (kg/m^2^), mean±SD	27.1 ± 4.2
Medical history		
	Myocardial infarction	19 (19.6%)
	Angioplasty	14 (14.4%)
	Stroke	9 (9.3%)
	Coronary bypass surgery	3 (3.1%)
Type of MI		
	STEMI	50 (51.5%)
	NSTEMI	47 (48.5%)
Management		
	Drug treatment	13 (13.4%)
	Coronary bypass surgery	5 (5.2%)
	Angioplasty	79 (81.4%)
	Bare-metal stent	55 (56.7%)
	Drug-eluting stent	24 (24.7%)
Biology		
	LDL-cholesterol level (g/L), mean±SD	1.28 ± 0.47
	HbA1C (%), mean±SD	7.5 ± 1.8
Cardiac	LVEF (%), mean±SD	53.7 ± 9.8

Data are n (%) unless indicated.

STEMI, ST-elevation MI; NSTEMI, non-ST-elevation MI; HbA1C, glycated hemoglobin; LDL, low-density lipoprotein; LVEF, left-ventricular ejection fraction

**Table 2:** Proportion of adherence to recommended targets according to death and survival at 1 year and 6 years.

Target’s achievement post MI on the previous evaluation	1 year post MI			6 years post MI		
	Death N = 5 (%)	Survival N = 91(%)	OR (IC95%), p value	Death N = 11 (%)	Survival N = 62 (%)	OR (IC95%), p value
BASI treatment	4 (80)	67 (74)	1.43 (0.12–13.46), p = 0.753	5 (45)	34 (55)	0.68 (0.19–2.49), p = 0.567
LDL cholesterol<1g/L	2 (40)	39/81 (48)	0.72 (0.11–4.53), p = 0.724	6 (55)	45 (73)	0.45 (0.12–1.68), p = 0.237
HbA1C<6.5%	1/3 (33)	10/21 (48)	0.55 (0.01–7.03), p = 0.646	2/6 (33)	9/15 (60)	0.33 (0.05–2.43), p = 0.279
No smoker	4 (80)	82 (90)	0.44 (0.04–4.37), p = 0.482	10 (91)	57 (92)	0.88 (0.09–8.32), p = 0.909
Achievement of all targets	1 (20)	9 (10)	2.28 (0.23–22.65), p = 0.482	2 (18)	19 (31)	0.50 (0.10–2.55), p = 0.407

BASI, treatment including a beta-blocker, a platelet aggregation inhibitor, a statin and an ACEI/ARB; LDL, low-density lipoprotein; MI, myocardial infarction

### Follow-up

The treatment was adapted by the general practitioner on the advice of the cardiologist for 75% (73/97) patients, by both the general practitioner and the cardiologist for 12% (12/97) patients and by the general practitioner alone for 5% (5/97) patients. There was no modification of treatment for 7% (7/97) patients.

### Non-fatal cardiovascular events

At 6 months, 43/97 (44.3%) patients had a non-fatal cardiovascular event: 12 had scheduled angioplasty, 10 urgent angioplasty, 10 another MI, 9 acute cardiac failure and 2 coronary bypass surgery. At 1 year, 17/91 (18.7%) patients had a non-fatal cardiovascular event: 4 more patients had urgent angioplasty, 4 another MI, 3 acute cardiac failure, 3 coronary bypass surgery, 2 scheduled angioplasty and 1 a stroke. At 6 years, 18/62 (29.0%) patients had at least one non-fatal cardiovascular event: acute coronary syndrome (9 events), peripheral artery disease (5 events), acute cardiac failure (5 events), heart rhythm disorders (3 events) and cerebrovascular disease (2 events). Nine of the 18 (50.0%) had more than one non-fatal cardiovascular event and 4/18 (22.2%) had not achieved the recommended therapeutic treatment.

### Achievement of recommended targets

**Fig 2:**
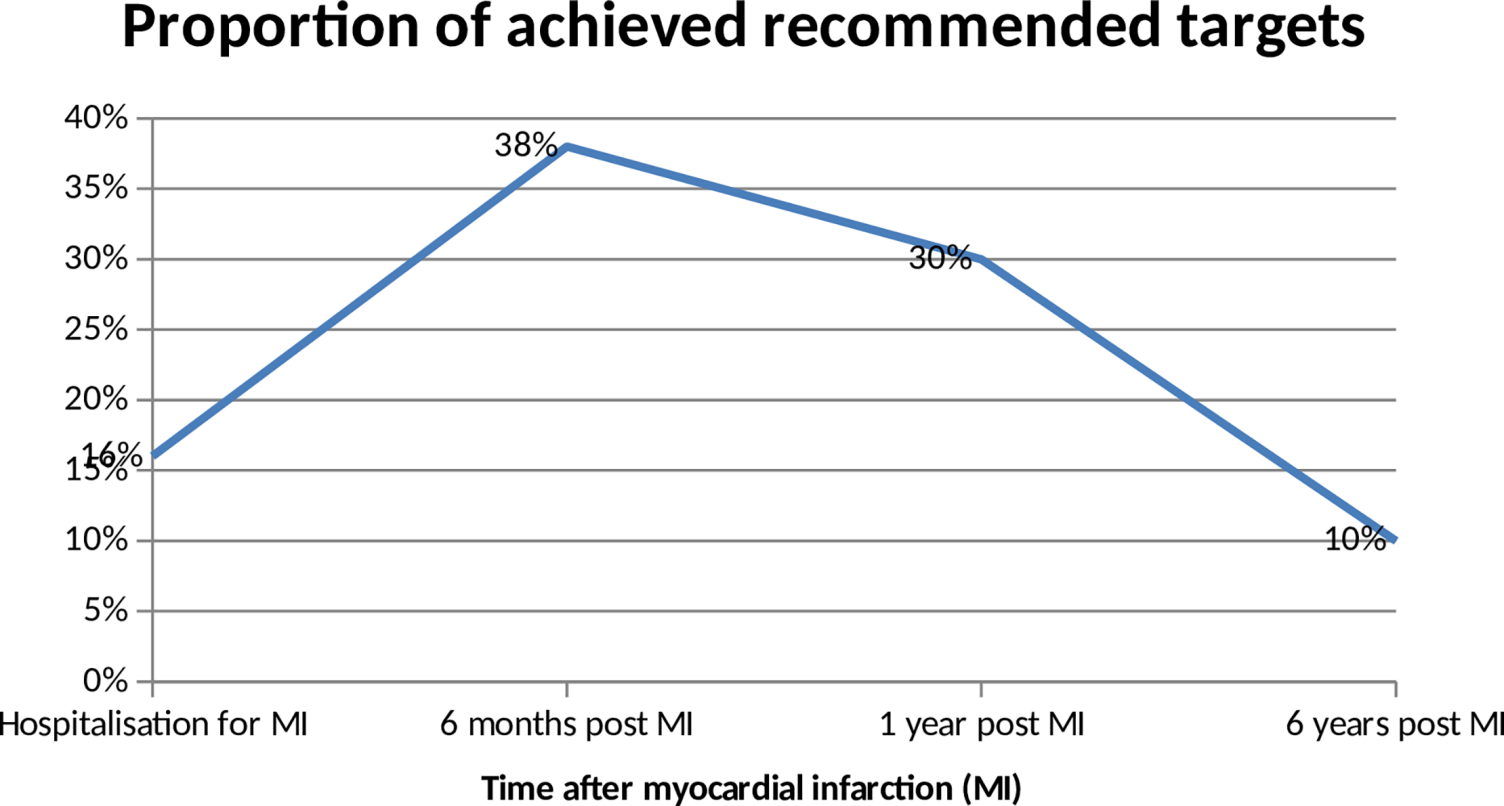
Proportion of achieved recommended targets over time.

A total of 10% (6/62) patients achieved the recommended targets in terms of prescriptions of treatment (i.e. prescription of a complete BASI treatment), risk factors (i.e. LDL-cholesterol<1g/L, blood pressure<140/90 mmHg and HbA1C<6.5% for diabetic patients) and lifestyle targets (no smoking). Evolution of proportions of recommended targets’ achievement over time is presented in Fig 2. Prescriptions of recommended treatment and risk factors targets are presented in Table 3. Data on treatment prescriptions in Table 3 concerned only the class of molecule prescribed without considering the type of molecule and dose. If we consider the prescription of recommended molecules and dose for ACEIs/ARB and betablockers at 6 months, 1 year and 6 years, 25% (24/97), 31% (28/91) and 16% (8/50) of patients, respectively, were receiving a recommended ACEIs/ARB molecule at an optimal dose, and 35% (34/97), 32% (29/91) and 23% (11/47) a recommended beta-blocker molecule at an optimal dose. Among the 12 patients who did not receive any beta-blockers, reasons for their non-prescription were mentioned for 6: bradycardia < 45 beats/minute (n = 2), chronic obstructive pulmonary disease (n = 2), peripheral artery disease (n = 1) and Raynaud’s disease symptoms (n = 1). For lifestyle targets at 6 years, 27/62 patients (44%) declared having performed a cardiac rehabilitation program in a specific centre. Among the 27 patients, 9 (33%) declared performing a recommended physical activity such as biking, running, swimming or walking at least 2 hours per week versus 3/35 (8.5%) who did not perform a cardiac rehabilitation program.

**Table 3:** Achievement of recommended treatment and risk factors objectives at hospital discharge and 6 months, 1 year and 6 years.

Objectives		Hospital discharge n = 97	6 months n = 97	1 year n = 91	6 years n = 62
Treatment objectives					
	BASI	72 (74%)	65 (67%)	44 (48%)	42 (68%)
	Beta-blockers	87 (90%)	82 (85%)	76 (84%)	50 (81%)
	Aspirin	96 (99%)	92 (95%)	76 (84%)	50 (81%)
	Clopidogrel	93 (96%)	89 (92%)	73 (80%)	22 (35%)
	ACEI/ARB	82 (85%)	76 (78%)	71 (78%)	53 (86%)
	Statin	95 (98%)	90 (93%)	82 (90%)	53 (86%)
Risk factors objectives					
	LDL cholesterol, g/L				
	<1	30 (31%)	59/86 (69%)	60/85 (71%)	43/57 (70%)
	<0.8	12 (12%)	45/86 (52%)	36/85 (42%)	23/57 (37%)
	<0.7	4 (4%)	28/86 (33%)	29/85 (34%)	14/57 (23%)
	HbA1C <6.5%	2/20 (10%)	9/20 (45%)	(42.9%)	4/13 (31%)
	Blood Pressure <140/90,mmHg	/	/	/	57 (92%)
	No smoking	74 (76%)	87 (90%)	85 (93%)	56 (90%)
**Total = achievement of all objectives**		**13 (13%)**	**35 (38%)**	**27 (30%)**	**6 (10%)**

ACEI/ARB, angiotensin-converting enzyme inhibitor/angiotensin receptor blocker; BASI, treatment including a beta-blocker, a platelet aggregation inhibitor, a statin and an ACEI/ARB; LDL, low-density lipoprotein.

### Factors affecting achievement of recommended objectives

Performing a cardiac rehabilitation program was associated with achieving all recommended targets (OR = 22.07 [95% CI 1.18–411.43]; p< 0.038). Achievement of recommended targets depending on cardiac rehabilitation is presented in Table 4. No other factor affected achievement of recommended targets.

**Table 4:** Achievement of recommended treatment and risk factors objectives at 6 years according to cardiac rehabilitation.

Targets’ achievement at 6 years post MI N = 63	Patients with a cardiac rehabilitation N = 27 (%)	Patients without any cardiac rehabilitation N = 36 (%)	OR (IC95%)	P value
BASI treatment	24 (89)	18 (50)	8.00 (2.04–31.37)	0.003*
LDL cholesterol<1g/L	20 (74)	22 (61)	1.82 (0.61–5.41)	0.283
HbA1C<6.5% (n = 13 diabetic patients)	4/6 (67)	0/7 (0)	27 (1.04–698)	0.047*
Blood pressure<140/90mmHg	1 (4)	2 (6)	0.65 (0.06–7.61)	0.734
No smoker	24 (89)	33 (92)	0.73 (0.13–3.92)	0.711
Achievement of all targets	6 (22)	0 (0)	22.07 (1–411)	0.038*

*P value<0.05

BASI, treatment including a beta-blocker, a platelet aggregation inhibitor, a statin and an ACEI/ARB; LDL, low-density lipoprotein; MI, myocardial infarction

## Discussion

### Significance of the results

A minority of patients achieved all the recommended targets in terms of therapy, lifestyle and cardiovascular risk factors. Guidelines on secondary prevention after a myocardial infarction evolved since our work. For example, LDL-cholesterol targets were interpreted according to European recommendations [3]. Although since 2013, they differed from the US recommendations [13], which advocated a targeted dose of statins, most patients achieved neither the European nor US recommendations. HbA1C targets in diabetic patients also changed since 2013, with a targeted HbA1C between 7% and 9% depending on the patient’s condition. In Table 3, we present the objective of HbA1C <6.5% at 6 years after the MI for comparison with the 1-year results; however, if we consider a new target of HbA1C <7%, 69% (43/62) patients achieved this target at 6 years post-MI. However, this HbA1C objective was difficult to interpret according to the new guidelines, because patients with HbA1C between 7% and 9% could receive appropriate management in case of short life expectancy or health comorbidities. For smoking cessation, the persistence of smoking habits in 6 (9.7%) patients at 6 years would require a specific management strategy to decrease that rate. Those strategies could involve psychosocial interventions with recommended nicotine replacement therapies with proved efficacy on decreasing smoking in a post-MI population [3].

We chose to collect our data through general practitioner’s questionnaires at 1 year and 6 years only. We tried to minimize the risk of desirability bias in observational studies, especially those involving the following of guidelines [14]. Therefore, we did not add intermediate results that would have interfered with the usual way of general practitioners’ care. Data on physical activity were reported only 6 years after the MI and were based on patient declarations, which could indicate a desirability bias that would overestimate the real physical activity. Additionally, we did not collect data on dietary habits for the same desirability bias reason.

Tools measuring the number of footsteps or the amount of spent energy per day would improve physical activity assessment in further studies and could improve adherence to drugs and lifestyle changes [15].

### Clinical implications and perspectives

Despite some limitations (especially a small sample size and potential declaration bias for the physical activity reported by patients), this study is original because it was performed in a general practice setting. Data were collected from the general practitioner who worked in collaboration with a cardiologist for the majority of treatment adaptations. This finding illustrates the importance of increasing research on the collaboration between professionals outside the hospital and its efficacy in terms of recommendations after hospital discharge, as outlined in the updated European guidelines [16]. The long-term follow-up of the FAST-MI cohort will also bring important information on post-MI morbidity and mortality, as it already showed the effectiveness of the cardiac rehabilitation 5 years after MI [17].

Cardiac rehabilitation appeared to be the only factor that significantly improved the number of achieved recommended therapeutic objectives. Cardiac rehabilitation was already associated with improved prognosis a 6 months after MI [8]. However, our small sample size and missing data may have led to missing some other potential predictors of achieving recommended therapeutic objectives, i.e. gender, comorbidities or social conditions [8]. Only 27/62 of patients (44%) declared having performed a cardiac rehabilitation program. Cardiac rehabilitation was performed in Bois Gibert, a monocentric center associated with the university hospital of Tours. The cardiac rehabilitation program was personalized and consisted in a daily physical training (walking, biking, running and/or swimming) associated with a dietary monitoring and therapeutic education performed by a multidisciplinary team with physicians, psychologists, nurses, physiotherapists and dieticians. Performing such a program could be a good way to decrease post-MI morbidity and mortality, even 6 years after a MI. Despite data for patients on reasons for non-prescription of the recommended treatment, the reasons for non-optimization of the treatment dose or molecule should be investigated in further studies.

## Conclusions

Cardiac rehabilitation could be a way to improve the achievement of recommended therapeutic objectives after MI. Larger studies need to be performed to confirm this finding, explore ways to improve close collaboration between general practitioners and cardiologists and consequently decrease the post-MI morbidity and mortality.

## Supporting information

S1 Appendix Recommended targets. ACEI, angiotensin-converting enzyme inhibitor; ARB, angiotensin receptor blocker; BASI, treatment including a beta-blocker, a platelet aggregation inhibitor, a statin and an ACEI/ARB; LDL, low-density lipoprotein.
(DOCX)
