# Supplementary material for: Patients’ adherence to optimal therapeutic, lifestyle and risk factors recommendations after myocardial infarction: Six years follow-up in primary care
Source: PLoS One. 2018 Sep 4;13(9):e0202986. doi: 10.1371/journal.pone.0202986 (PMC6122779; doi:10.1371/journal.pone.0202986)
Supplement: S1 Appendix — ACEI, angiotensin-converting enzyme inhibitor; ARB, angiotensin receptor blocker; BASI, treatment including a beta-blocker, a platelet aggregation inhibitor, a statin and an ACEI/ARB; LDL, low-density lipoprotein. (DOCX) [file pone.0202986.s001.docx]

**Appendix 1. Recommended targets**

| **Targets** | | **Criteria to achieve the target** |
| --- | --- | --- |
| Treatment Targets | |  |
|  | BASI | Adequate prescription of each of the following treatments |
|  | Beta-blockers | Prescription of a recommended Beta-blocker, i.e. metoprolol 200mg, propranolol 160mg, atenolol 50 to 100 mg, acebutolol 400mg or timolol 20mg |
|  | Aspirin | Prescription of aspirin |
|  | Clopidogrel | Prescription of clopidogrel |
|  | ACEI/ARB | Prescription of recommended ACEI/ARB, i.e. ramipril 10mg, captopril 75 to 150mg, trandolapril 4mg, lisinopril 10mg, zofenopril 60mg, valsartan 320mg, perindopril 10mg |
|  | Statin | Prescription of statin |
| Risk factors targets | |  |
|  | LDL cholesterol, g/L | LDL cholesterol<1g/L |
|  | HbA1C, % | HbA1C<6.5% |
|  | Blood Pressure, mmHg | Systolic blood pressure<140mmHg and diastolic blood pressure<90 mmHg |
|  | Smoking | No smoking or smoking cessation |

ACEI, angiotensin-converting enzyme inhibitor; ARB, angiotensin receptor blocker; BASI, treatment including a beta-blocker, a platelet aggregation inhibitor, a statin and an ACEI/ARB; LDL, low-density lipoprotein
